# Supplementary figures and images for: Transcriptome analysis reveals high tumor heterogeneity with respect to re-activation of stemness and proliferation programs
Source: PLoS One. 2022 May 19;17(5):e0268626. doi: 10.1371/journal.pone.0268626 (PMC9119523; doi:10.1371/journal.pone.0268626)

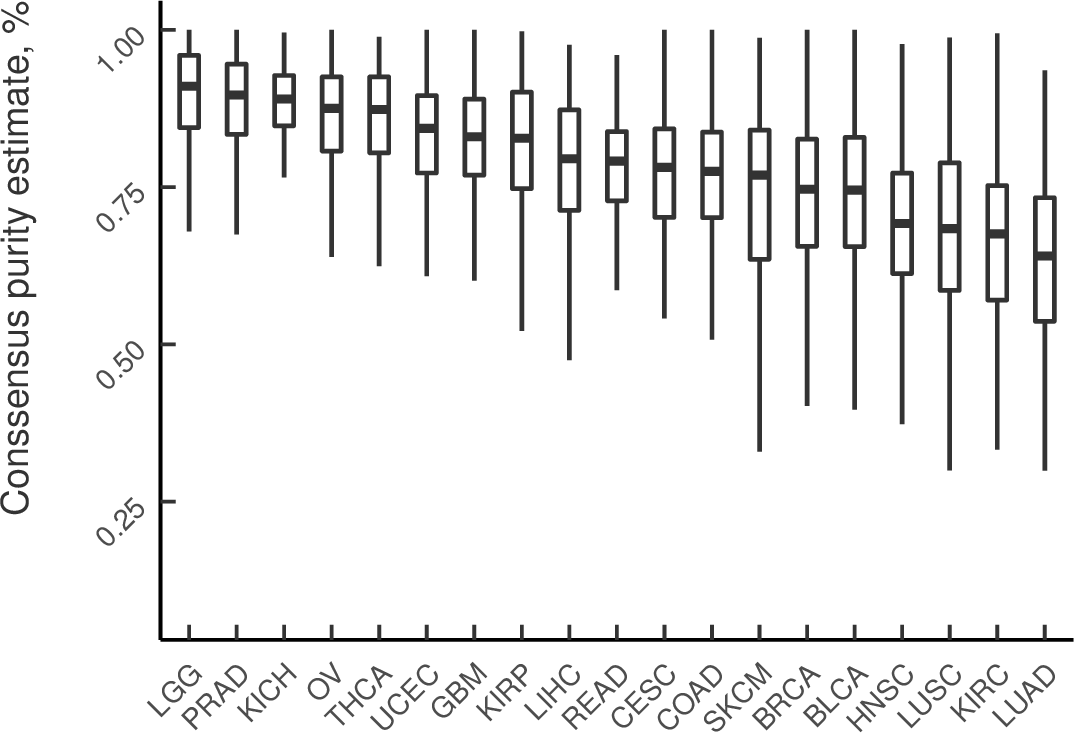

Supplement: S1 Fig — The tumor types on the x axis are listed by in the descending order by the mean CPE value. (TIF) [file pone.0268626.s001.tif]

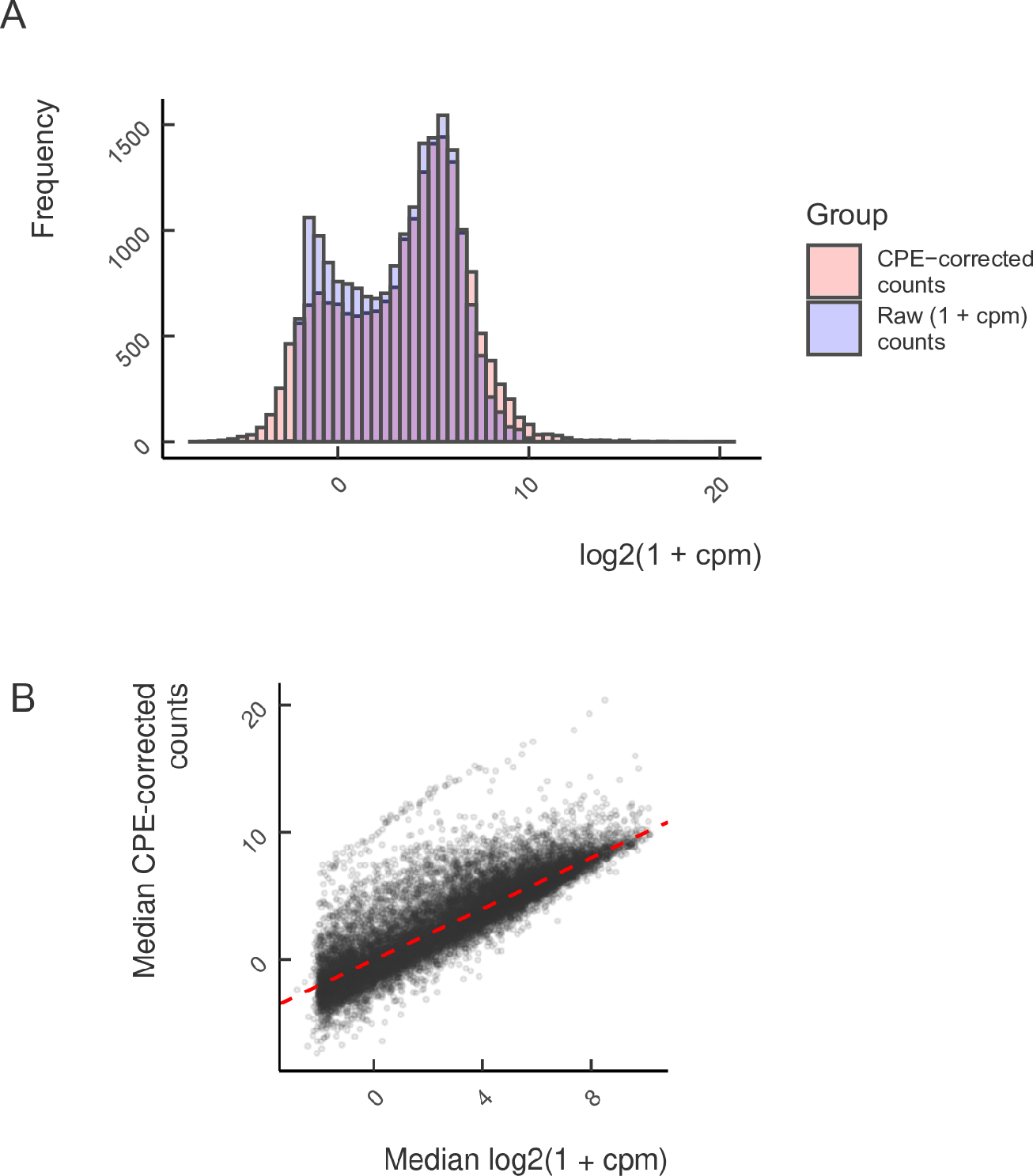

Supplement: S2 Fig — (A) The distribution of log2(1 + CPM), where CPM denotes median counts per million, for the raw and CPE-corrected gene expression values. The gene expression counts were first normalized by edgeR and then corrected for tumor purity (See Methods—Correction for tumor purity). (B) A scatter plot of CPE-corrected vs. raw log2(1 + CPM). Each point represents a gene. (TIF) [file pone.0268626.s002.tif]

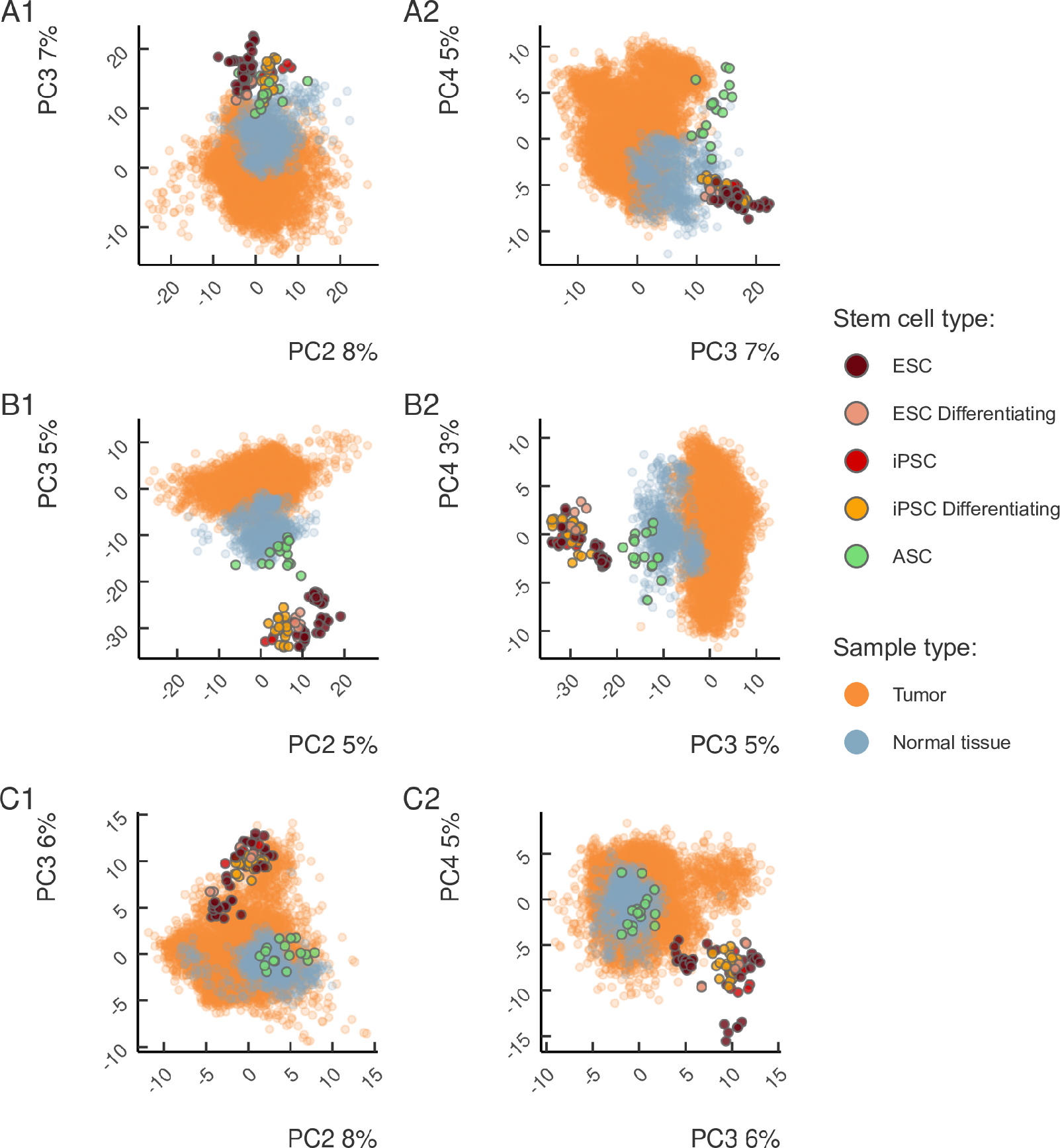

Supplement: S3 Fig — Panels (A), (B), and (C) correspond to the stemness, proliferation, and EMT-MET signatures, respectively. (TIF) [file pone.0268626.s003.tif]

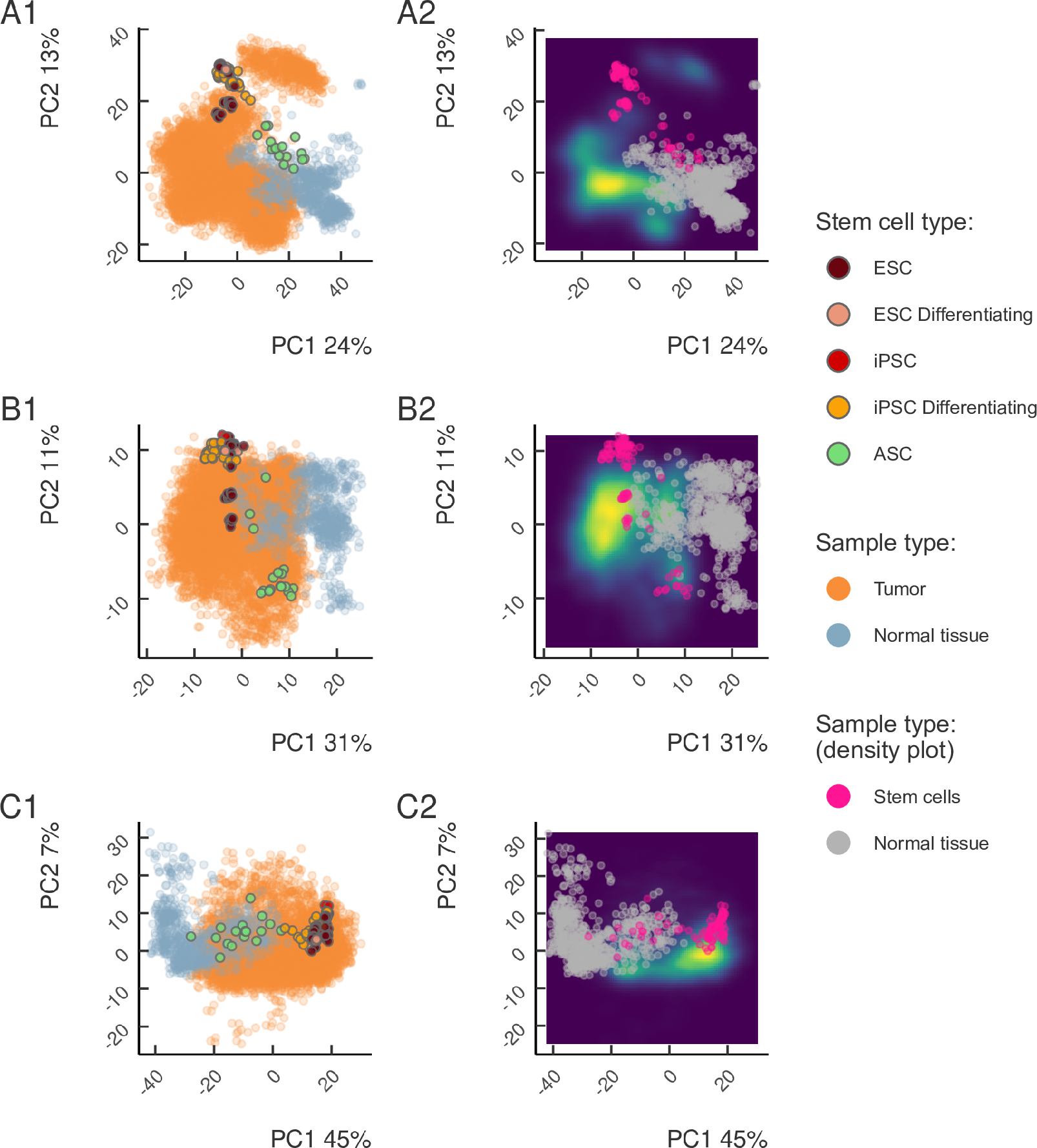

Supplement: S4 Fig — (TIF) [file pone.0268626.s004.tif]

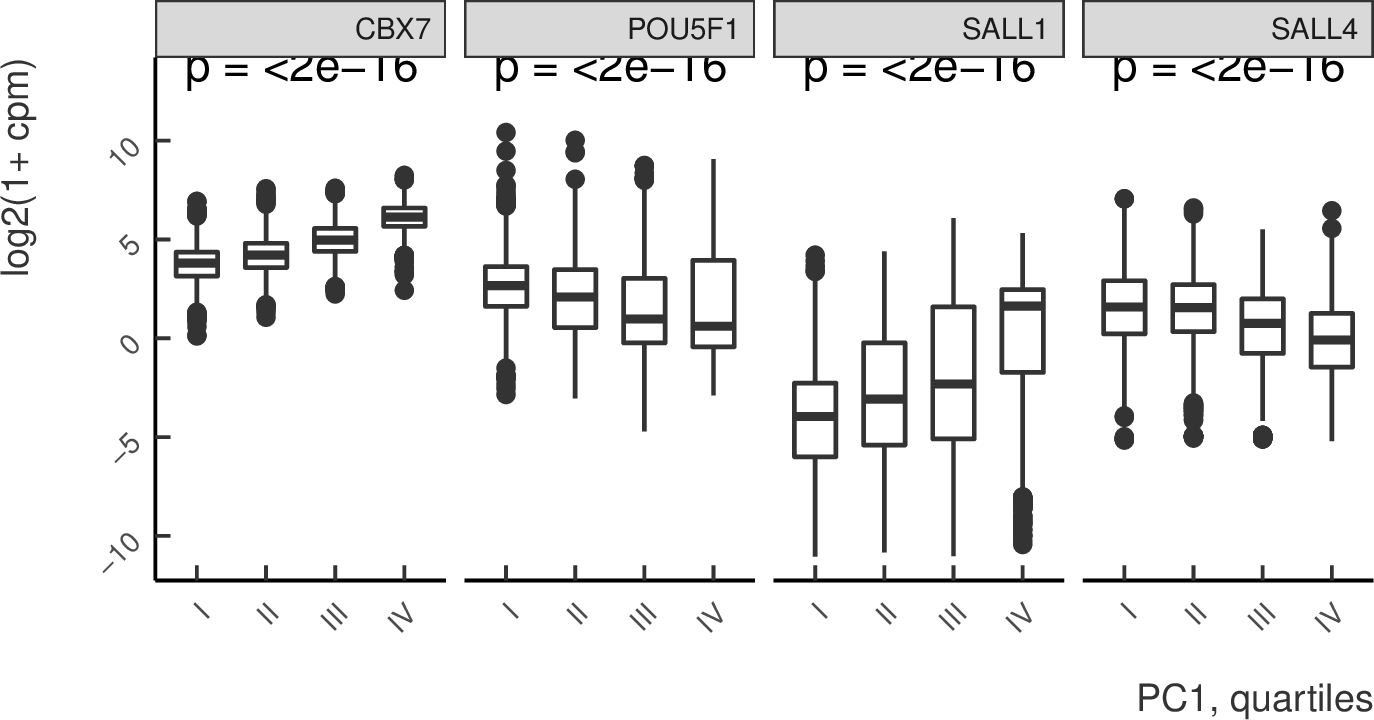

Supplement: S5 Fig — Samples were stratified into quartiles according to PC1. (TIF) [file pone.0268626.s005.tif]

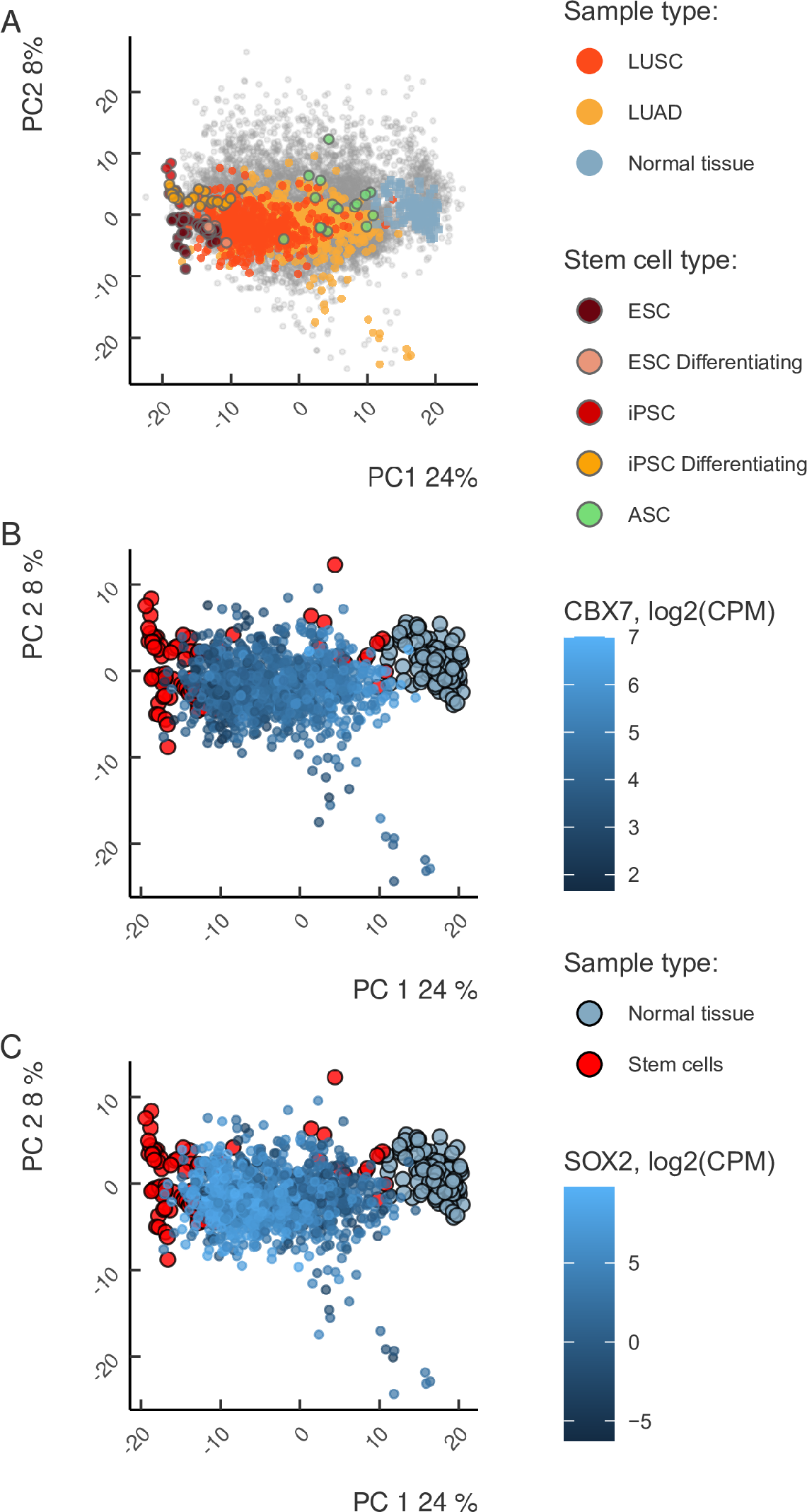

Supplement: S6 Fig — All tumor samples except for LUSC and LUAD are colored gray. The expression of CBX7 drops (B), and the expression of SOX2 increases (C) in tumors on the way from normal samples to iPSCs and ESCs. (TIF) [file pone.0268626.s006.tif]

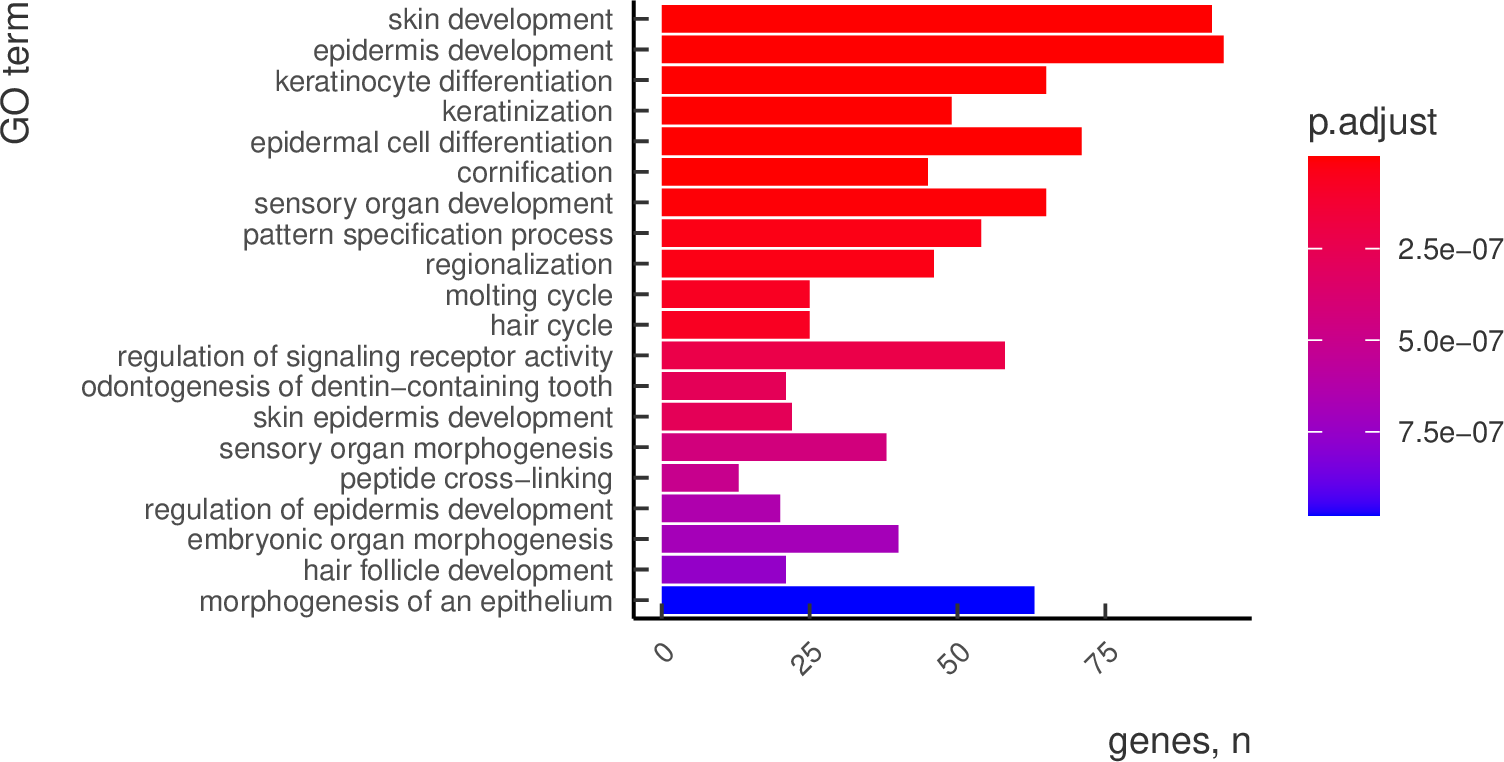

Supplement: S7 Fig — (TIF) [file pone.0268626.s007.tif]

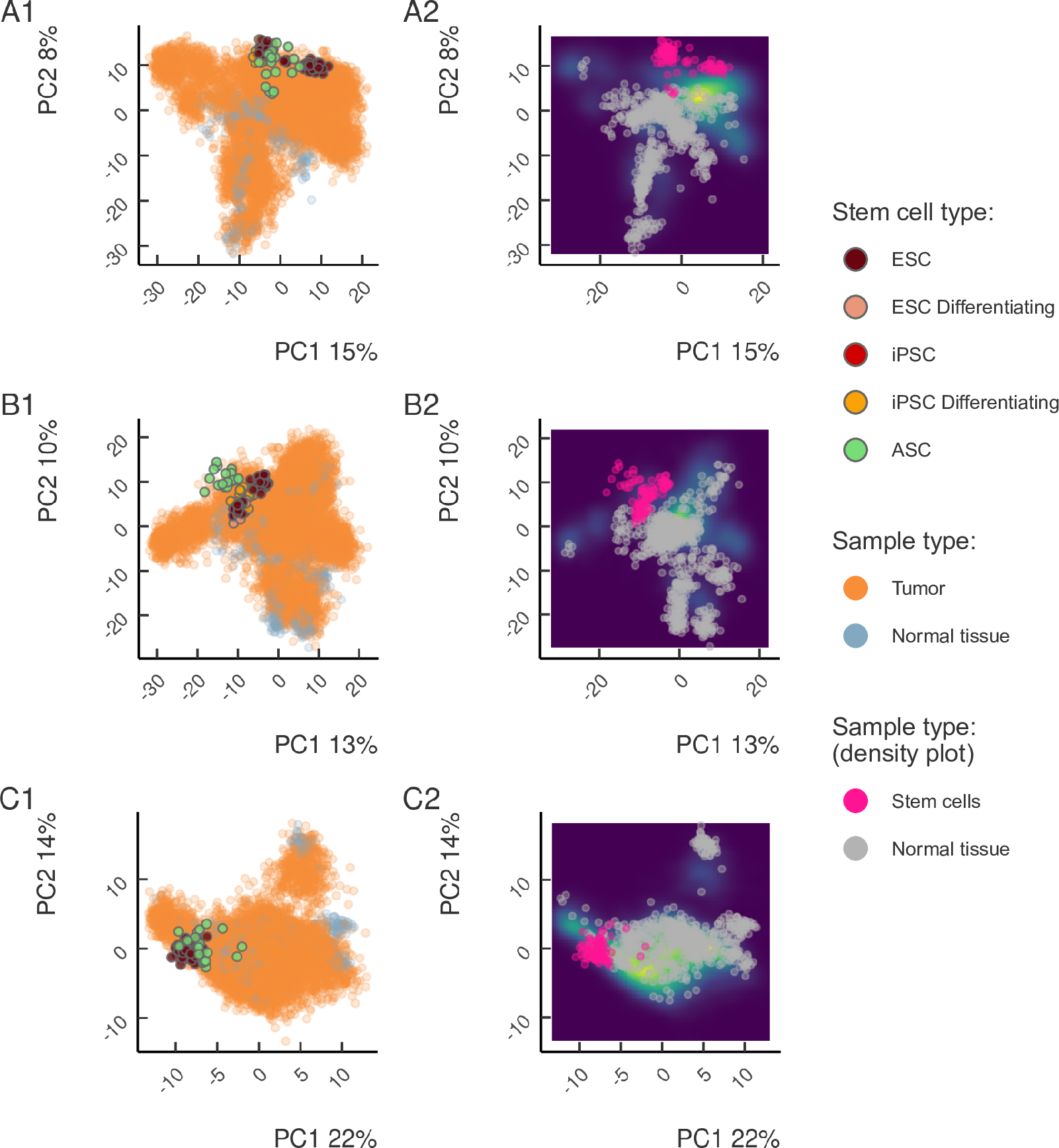

Supplement: S8 Fig — (TIF) [file pone.0268626.s008.tif]

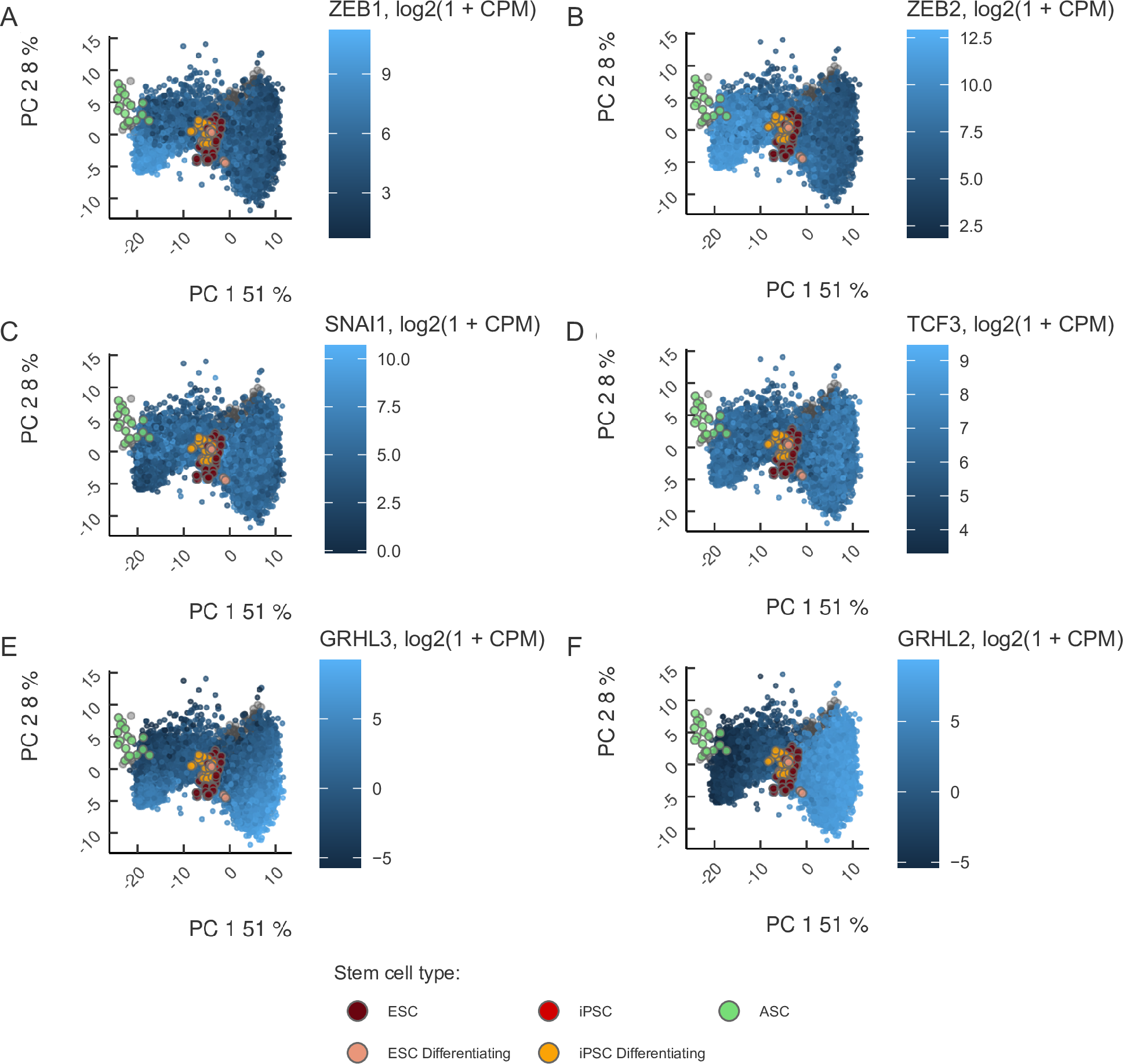

Supplement: S9 Fig — Normal tissues are shown as gray background. Stem cells are colored as in Fig 2. (TIF) [file pone.0268626.s009.tif]

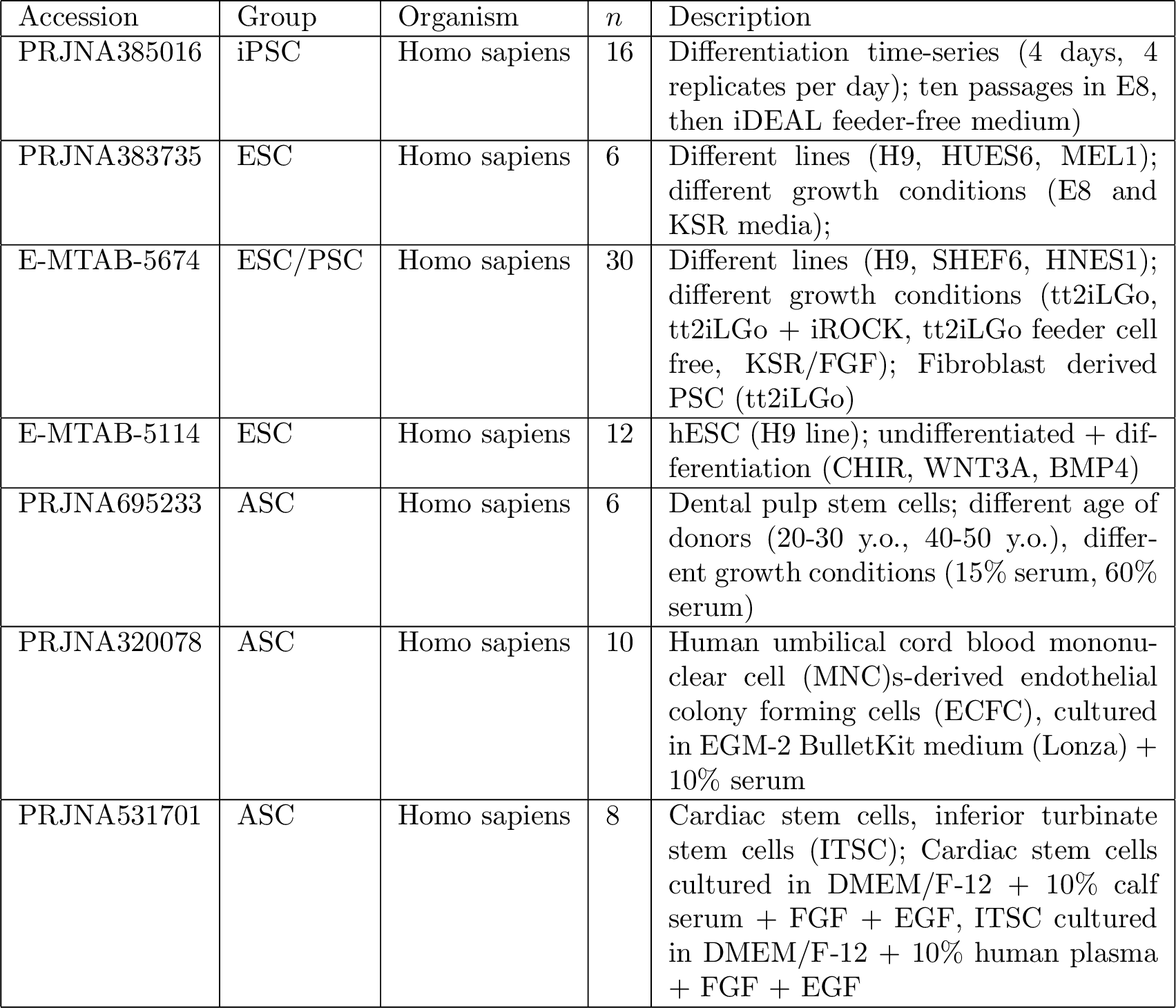

Supplement: S1 Table — (TIF) [file pone.0268626.s010.tif]

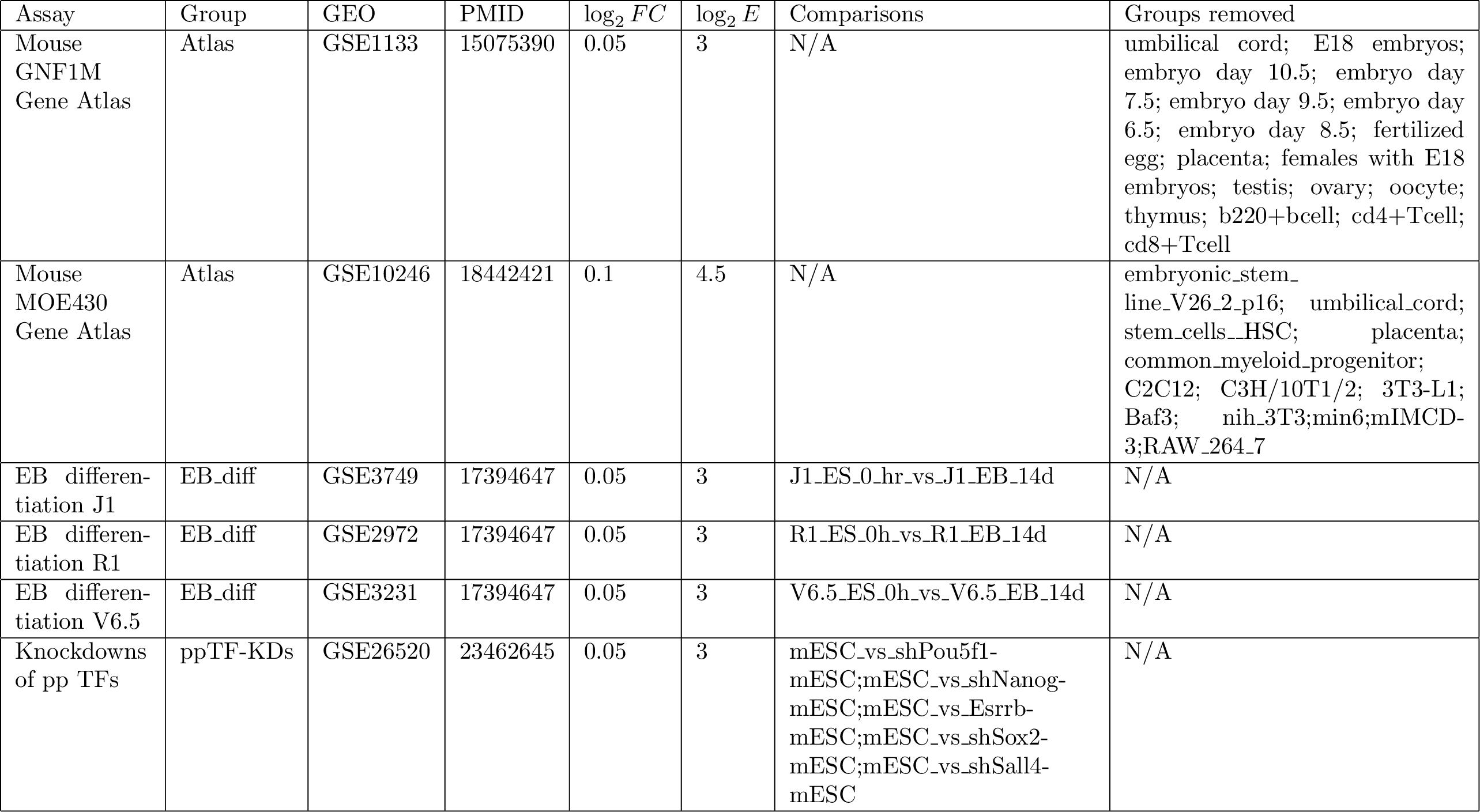

Supplement: S2 Table — Additional information is reported in Methods (see Signature gene sets section for details). log2 FC denotes log-fold-change threshold (Treatment vs Control); log2 E denotes log gene expression level threshold (Treatment + Control); Comparisons denotes the list of comparisons for differential gene expression analysis. (TIF) [file pone.0268626.s011.tif]
